# Supplementary material for: Failure mechanisms of single-crystal silicon electrodes in lithium-ion batteries
Source: Nat Commun. 2016 Jun 14;7:11886. doi: 10.1038/ncomms11886 (PMC4911629; doi:10.1038/ncomms11886)
Supplement: Supplementary Information — Supplementary Figures 1-5, Supplementary Notes 1-2, Supplementary Methods and Supplementary References. [file ncomms11886-s1.pdf]

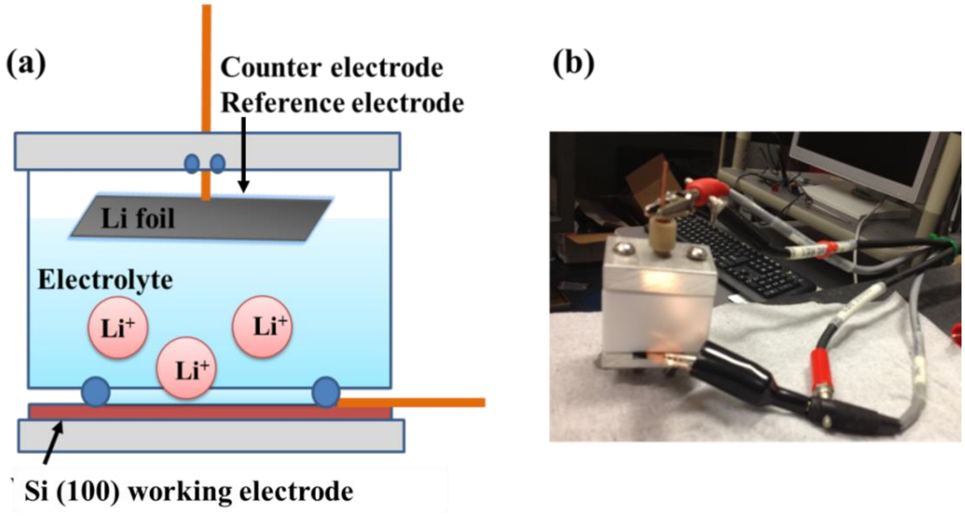

Supplementary Figure 1. (a) Schematic of the electrochemical reaction cell and (b) photograph of the experimental set-up used for electrochemical testing.

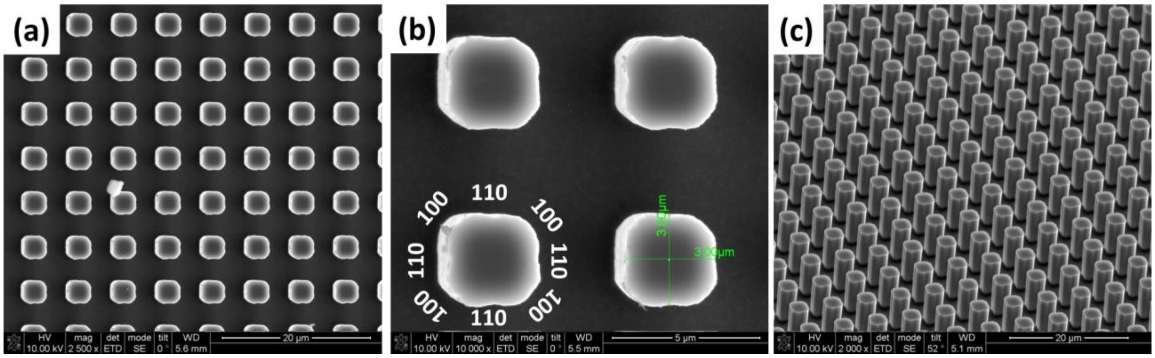

Supplementary Figure 2. Pristine Si micropillars fabricated on a Si(100) wafer by photolithography: (a, b) plane view and (c) 52º tilt angle view.

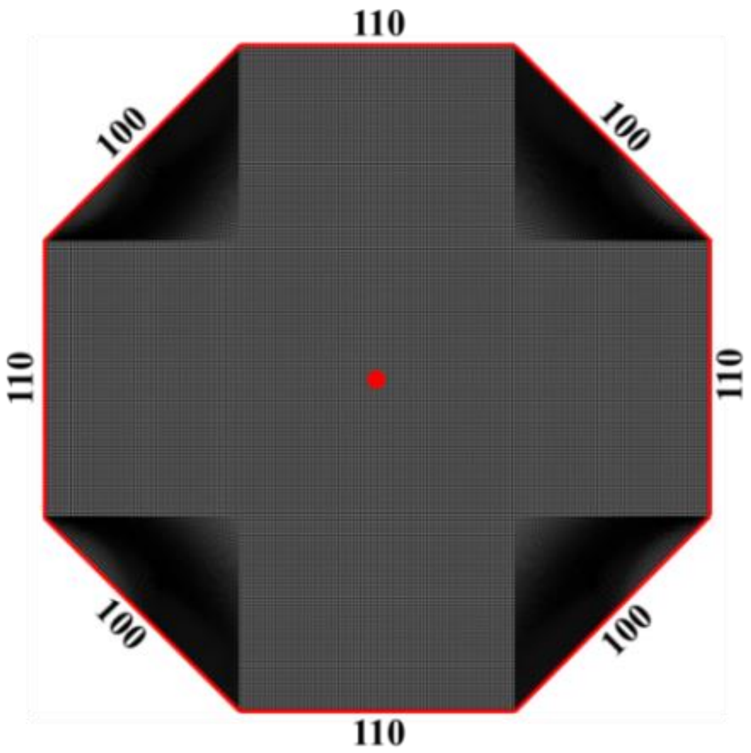

Supplementary Figure 3. Plane-strain FEM model of a Si micropillar “unit cell” with four <110> edges and four <100> edges.

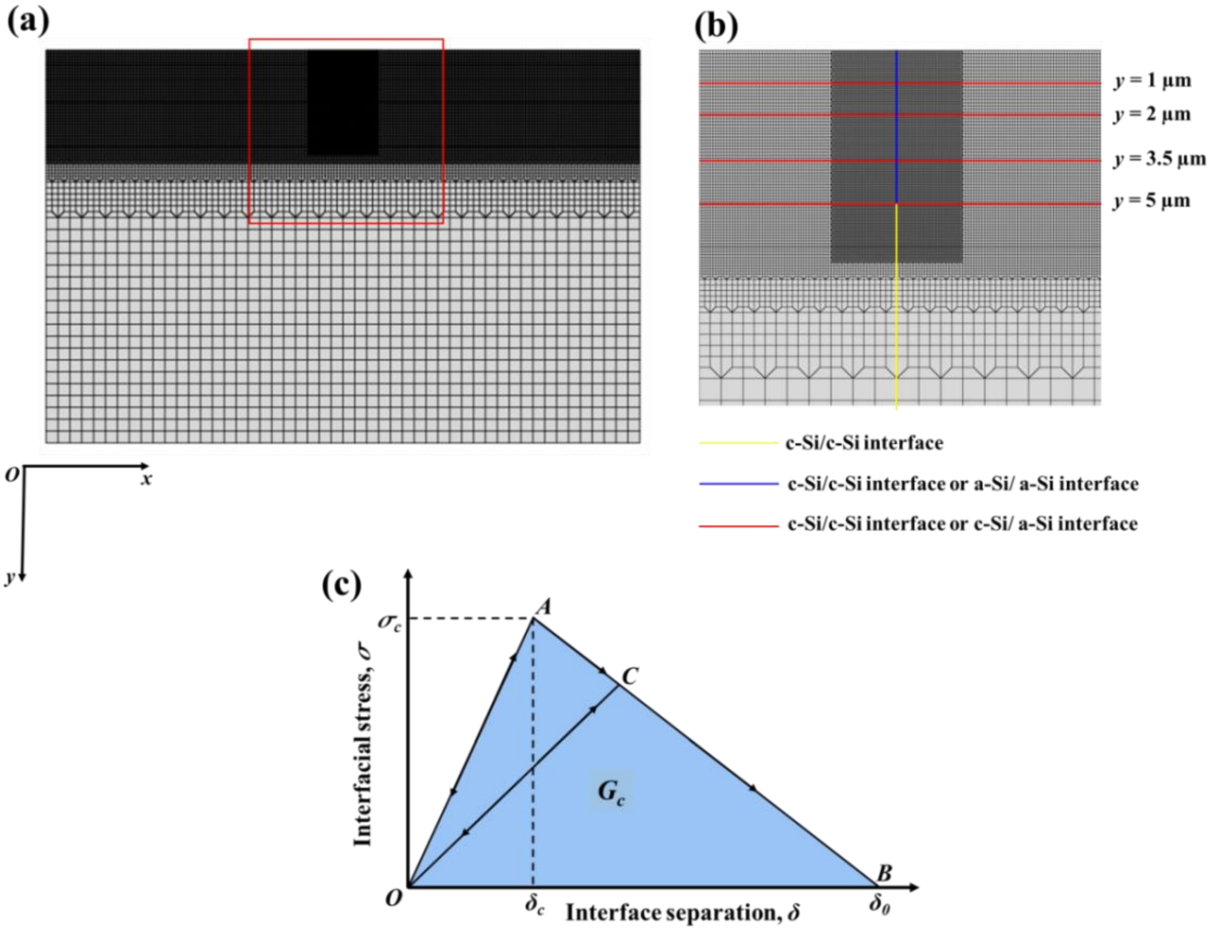

Supplementary Figure 4. (a) Plane-strain FEM model of a Si electrode. The left and right boundaries are constrained in the *x*-direction, while the bottom boundary is constrained in the *y*-direction. (b) Magnified view of the FEM mesh in the vicinity of potential cohesive interfaces. The yellow line represents the c-Si/c-Si interface, the blue line represents the c-Si/c-Si interface initially, which later becomes a-Si/a-Si interface as the lithiation depth increases, while the red line represents the c-Si/c-Si interface initially, which later changes to a-Si/c-Si interface with increasing lithiation depth. (c) Schematic of a bilinear cohesive zone model describing the relation between interfacial stress σ and interface separation δ. A surface separation greater than δ_c leads to crack initiation and propagation, accompanied by a decrease in both cohesive strength σ_c and interface work of adhesion (fracture energy) G_c until complete failure ($\delta = \delta_0$).

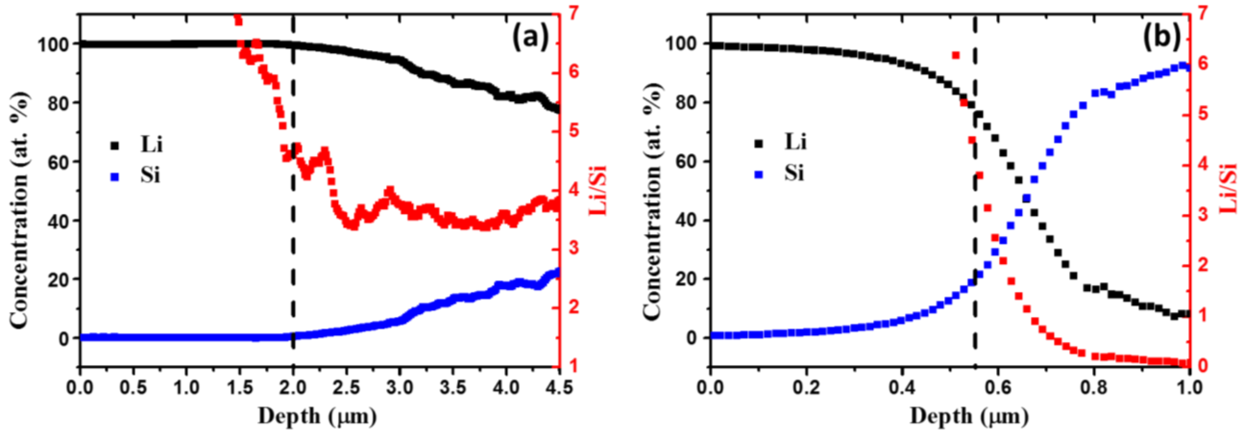

Supplementary Figure 5. Depth profiles of Li, Si, and Li/Si ratio through the thickness of a lithiated Si(100) electrode obtained from SIMS measurements: (a) reference $Li_{15}Si_4$ sample obtained by discharging a Si(100) electrode at 5 mV for 2 days and (b) half-cycle lithiated Si(100) electrode obtained by linear sweeping from the OCP to 5 mV for a scan rate of 0.1 $mV \cdot s^{-1}$. Due to the different sensitivity factors of each species, the Li/Si ratio was calibrated to the bulk composition of the reference sample.

Supplementary Note 1

Parameters of cohesive zone model

The present FEM model contains three types of interfaces, i.e., c-Si/c-Si, a-Si/c-Si, and a-Si/a-Si. For the virtual c-Si/c-Si interface we used σ_c and G_c of bulk c-Si. The tensile strength of single-crystal silicon varies between 1 and 20 GPa, depending on sample size, type, and preparation and testing method.[5] Thus, in the present study, we used $\sigma_c^{\text{c-Si}} = 5$ GPa. The measured K_{IC} and the fracture energy $G_c = K^2_{IC}/E$ are in the ranges of 0.7–1.3 MPa·m$^{1/2}$ and 2.7–9.2 J·m^{-2}, respectively. Consequently, in the present study, we used $G_c^{\text{c-Si}} = 9$ J·m^{-2}.

Similarly, for the σ_c and G_c of the a-Si/a-Si interface we used the properties of lithiated bulk silicon. A recent nanoindentation study[1] has shown that the yield strength of lithiated silicon is in the range of 0.43–3 GPa. In present study, we used $\sigma_c = 0.25$–1.5 GPa, which is within the reported range.

A direct measurement of σ_c and G_c of the a-Si/c-Si interface is almost impossible, because it requires accurate probing of the material interface with atomic resolution. Therefore, we used estimates from a first-principle MD simulation study,[6] which show that G_c varies in the range of 0.1–0.3 J·m^{-2}, depending on lithium concentration. Specifically, G_c is equal to ~0.1 and ~0.3 J·m^{-2} for high and low lithium concentration, respectively. Because of the different definition of G_c in the present study, the value must be doubled;[6] therefore, we used $G_c^{\text{a-Si/c-Si}} \approx 0.2$–$0.6$ J·m^{-2}. Due to the linear distribution of the lithium concentration through the electrode thickness, the lithium concentration is low in the a-Si close to the a-Si/c-Si interface and $G_c^{\text{a-Si/c-Si}}$ is close to 0.6 J·m^{-2}. In the present model, we chose to be more conservative by setting $G_c^{\text{a-Si/c-Si}} = 1$ J·m^{-2}, which represents an upper bound condition and ensures enough margin for simulating interfacial delamination. As evidenced from Fig. 4, the lower the $G_c^{\text{a-Si/c-Si}}$ the higher the likelihood of crack deflection at the bimaterial interface. Lastly, we used $\sigma_c = 1.5$ GPa, which is the lowest value for lithiated silicon with low lithium concentration and single-crystal silicon.

Once σ_c and G_c are determined, δ_0 can be calculated by $\delta_0 = 2G_c/\sigma_c$. If we take the c-Si/c-Si interface as an example ($\sigma_c = 5$ GPa and $G_c = 9$ J·m^{-2}), we find $\delta_0 = 3.6$ nm. δ_c represents the critical stretch (elongation) or the elastic limit, and is determined by the critical strain $\varepsilon = \sigma_c/E$ and the local mesh size a, i.e., $\delta_c = a\ \varepsilon = a\ \sigma_c\ /E$. Since the elastic modulus of c-Si used in the present study is $E = 185$ GPa and the local mesh size in the FEM model is 50 nm, it follows that $\delta_c = 1.35$ nm.

In this study, both $G_c^{\text{c-Si}}$ and $G_c^{\text{a-Si/c-Si}}$ were selected from the upper range of the reported values. This in turn gives a reasonable estimation of the interface-to-bulk work of adhesion ratio, which is the critical parameter that determines the crack trajectory when the crack propagates toward the bimaterial interface (Fig. 4).

In the first cycle, the red line ($y = 1$ μm) represents the a-Si/c-Si interface. The cohesive interface above the a-Si/c-Si interface (part of the blue line) is a-Si/a-Si interface, whereas the cohesive interface below the a-Si/c-Si interface (part of the blue, yellow, and red lines) is c-Si/c-Si interface. As the lithiation depth increases with the lithiation/delithiation cycles, the a-Si/c-Si interface moves to the second ($y = 2$ μm), third ($y = 3.5$ μm), and fourth ($y = 5$ μm) red line and the cohesive interface above and below the a-Si/c-Si interface is accordingly updated.

Supplementary Note 2

SIMS measurements

Two samples were examined with SIMS: a reference sample obtained by discharging a Si(100) electrode at 5 mV for 2 days (it has been reported[7] that these discharging conditions lead to the formation of $Li_{15}Si_4$) and a half-cycle lithiated Si(100) sample obtained by a linear sweep from the open current potential (OCP) to 5 mV at a rate of 0.1 $mV \cdot s^{-1}$. Supplementary Fig. 5 shows depth profiles of Li, Si, and Li/Si ratio obtained with SIMS. The vertical dashed line at Li/Si = 3.75 indicates the boundary between the solid electrolyte interface (SEI) and lithiated Si. The significantly higher Li/Si ratio near the surface is attributed to Li trapped in the SEI and residual electrolyte which is almost free of Si. The Li/Si ratio reaches a steady-state of ~3.75 at depths ≥2 μm (Supplementary Fig. 5a). This region is identified as the $Li_{15}Si_4$ phase. The 3.75 value was used to calibrate the Li/Si ratio (Supplementary Fig. 5b). The results clearly show a higher Li concentration at the electrode surface than the bulk and a fairly linear Li concentration distribution through the lithiated Si region. Therefore, a linear distribution of Li concentration was used in all numerical simulations.

Supplementary Methods

Electrochemical experiments

Electrochemical experiments were performed in a single-compartment Teflon cell shown in Supplementary Fig. 1. An electrolyte consisting of 1 M lithium hexafluorophosphate ($LiPF_6$) in ethylene carbonate (EC)/diethyl carbonate (DEC) (EC/DEC = 1:2 v/v) (Novolyte Technologies, BASF) was used in all electrochemical tests. In the experiments aimed to study the effect of electrolyte additives on the electrode mechanical performance, 5% of vinylene carbonate (VC) or fluoroethylene carbonate (FEC) additive was added to the base electrolyte. Dimethyl carbonate (DMC) (>99.9%, HPLC grade, Sigma-Aldrich) was used as the rinsing solvent. The counter and reference electrodes were made of Li, whereas the working electrodes were 2×2 cm^2 pieces cut from Si(100) wafers with or without vertically standing Si micropillars. The potentials reported here are referred to the Li/Li^+ redox couple. All the electrochemical tests were performed inside a glove box filled with Ar gas (H_2O and O_2 contents <10 ppm). A multi-channel potentiostat (Multistat 1480, Salartron Analytical) was used for potential/current control.

Electrode fabrication

In this study, p-type boron-doped Si(100) wafers (MTI) of 100 mm (4 in.) diameter and 0.001 Ω·cm electrical resistivity were used as electrodes. The native oxide film was removed by first treating with diluted 5% HF and then rinsing with ultra-pure water (18.2 MΩ·cm) for 2 min. Silicon micropillars were fabricated by dry etching the wafers using photolithography (Supplementary Fig. 2). The cross-sectional area and height of the pillars were precisely controlled to be 3×3 μm^2 and 8 μm, respectively. Before performing photolithography, the mask was aligned with two <110> notches at the wafer side. The pillars were fabricated with their {110} lateral surfaces exposed and their round corners in the (100) direction (Supplementary Fig. 2b).

Surface morphology characterization

After electrochemical treatment, the Si samples (both with and without micropillars) were rinsed in DMC to remove the residual electrolyte. The samples were then transferred to a scanning electron microscope (SEM) (JSM-6700F, JEOL) for imaging. The electrode microstructure was observed with a focused ion beam (Nova 600i Dual Beam, FEI). The cycled electrodes were cross-sectioned with a Ga^+ ion beam and observed with the SEM.

Secondary ion mass spectrometry

Secondary ion mass spectrometry (SIMS) measurements of the lithiated electrodes were obtained by Evans Analytical Group. The Cameca IMS 4f mass spectrometer was operated at a pressure of 2.67×10^{-7} Pa (2×10^{-9} torr). A 3 keV Cs^+ sputtering beam with 200×200 μm^2 sputtering area was used in all SIMS measurements. Elemental concentration depth profiles were obtained by analyzing the positively charged CsLi and CsSi secondary ions. The Cs^+ current was typically equal to 10 nA. The sputtering depth was determined from the depth of the sputtering craters measured with a profilometer. Data acquisition and post-processing analysis were performed with SIMSview software. The lithiated samples were sealed in a glove box and transferred to the SIMS spectrometer within 1 min.

Finite element modeling

The finite element method (FEM) code ABAQUS/Standard (6.14 version) was used to determine the in-plane stress and strain fields due to anisotropic lithiation/delithiation and to study crack initiation, propagation, deflection, and, eventually, delamination on a silicon electrode subjected to multiple lithiation/delithiation cycles. The unlithiated (crystalline) silicon (c-Si) was modeled as an isotropic elastic material with an elastic modulus of 185 GPa and a Poisson's ratio of 0.23. Although c-Si is not strictly an isotropic elastic material, because its elastic modulus may vary by 30% among different crystalline orientations, this variation is considered to be secondary due to the strong orientation-dependence of the lithium ion diffusion rate. The lithiated (amorphous) silicon (a-Si) was modeled as an isotropic elastic-perfectly plastic material. Depending on lithium ion concentration, the elastic modulus and yield strength of lithiated silicon vary in the range of 50–102 GPa and 0.5–3.0 GPa, respectively.[1]

Finite element model of a micropillar "unit cell"

Supplementary Fig. 3 shows the plane-strain FEM model of a Si micropillar with four {110} edges and four {100} edges of length equal to 2 μm, representing the four sides and four round corners, respectively, in the top-view SEM image shown in Supplementary Fig. 2b. The equilateral octahedral FEM model consists of 56,800 four-node linear isoparametric elements of uniform size equal to 0.02 μm. The total number of nodes is 57,201. All edges are exposed to lithium ion diffusion. The rate of lithium ion diffusion D depends on the crystal orientation (i.e., $D_{\{110\}} = 6.4\ D_{\{100\}} = 6.1\ D_{\{111\}}$).[2] In the present analysis, lithiation is modeled as a quasi-static diffusion process. As such, the stresses and strains are determined by the relative rate of lithium ion diffusion in different crystal orientations, not the absolute diffusion rate. By using a homogeneous initial temperature field and a constant thermal expansion coefficient, the ingression of lithium ions is simulated by a temperature front moving at different speeds in different directions. Thus, lithiation-induced volume expansion and associated stress field are equivalently represented by thermal expansion and thermal stress distribution.

To prevent rigid body movement in the simulations of the unconstrained micropillar "unit cell" (Fig. 2g), the center node of the FEM model (the red dot in Supplementary Fig. 3) was fully constrained against in-plane displacement and rotation. In the simulations of the constrained micropillar "unit cell" (Fig. 2h-i), the nodes at all edges (shown by red lines in Supplementary Fig. 3) were constrained against in-plane displacement and rotation, in order to simulate the constraint induced by the surrounding material.

Finite element model of electrode fracture

Supplementary Fig. 4a shows the plane-strain FEM model used to study crack initiation, propagation, deflection, and delamination through the thickness of the Si electrode. The model consists of 43,235 four-node linear elements comprising 46,018 nodes. The element size in the vicinity of the potential crack path is equal to 0.05 μm, gradually increasing to 4 μm in the region away from the lithiated region. The left and right boundaries of the mesh are constrained against displacement in the x-direction, while the bottom boundary is constrained against displacement in the y-direction. Multiple interfaces (the colored lines in Supplementary Fig. 4b) are modeled with a bilinear cohesive zone model, which can be used to simulate crack initiation and growth by allowing the interfaces to separate from each other.[3,4] Supplementary Fig. 4c shows a schematic of the bilinear traction-separation law of the cohesive interface, where σ is the interfacial stress (either normal or parallel to the interface), δ is the interface separation, σ_c is the cohesive strength, δ_c is the interface separation for crack initiation, δ_0 is the interface separation

for failure (i.e., delamination), and G_c is the interface work of adhesion (fracture energy), represented by the area (OAB), i.e.,

$$G_c = \frac{1}{2}\,\sigma_c\delta_0 \tag{S1}$$

In the present analysis, it is assumed that δ_c, δ_0, σ_c, and G_c are the same in both normal and shear directions. δ_0 is obtained in terms of σ_c and G_c (Eq. (S1)), whereas δ_c represents the critical separation at the interface corresponding to an interfacial stress equal to the cohesive strength σ_c and is obtained in terms of the critical strain $\varepsilon = \sigma_c/E$ and local mesh size a as

$$\delta_c = a \cdot \varepsilon = a(\sigma_c/E) \tag{S1}$$

Crack initiation and complete failure are, respectively, controlled by the following relations:

$$[\sigma_n^2 + \sigma_s^2]^{1/2} = \sigma_c \tag{S3}$$

$$G_n + G_s = G_c \tag{S4}$$

where subscripts n and s denote the normal and in-plane shear directions with respect to the crack plane, respectively.

Thus, the stress-separation law at the interface can be expressed as

$$\sigma = \begin{cases} \left(\frac{\delta}{\delta_c}\right)\sigma_c & (0 < \delta \le \delta_c) \\ \left(\frac{\delta_0-\delta}{\delta_0-\delta_c}\right)\sigma_c & (\delta_c < \delta < \delta_0) \\ 0 & (\delta \ge \delta_0) \end{cases} \tag{S5}$$

For $\delta \le \delta_c$, the interfacial stress increases linearly with surface separation, implying purely elastic stretching at the interface, while for $\delta_c < \delta < \delta_0$, the interfacial stress decreases linearly from σ_c to zero as the crack propagates. Crack growth leads to an unloading path (CO) which is different from the loading path (OA). Complete failure occurs when $\delta \ge \delta_0$, resulting in a locally stress-free interface.

Supplementary References

1. Berla, L. A., Lee, S. W., Cui, Y. & Nix, W. D. Mechanical behavior of electrochemically lithiated silicon. *J. Power Sources* **273,** 41-51 (2015).
2. Pharr, M., Zhao, K., Wang, X., Suo, Z. & Vlassak, J. J. Kinetics of initial lithiation of crystalline silicon electrodes of lithium-ion batteries. *Nano Lett.* **12,** 5039-5047 (2012).
3. Tvergaard, V. & Hutchinson, J. W. Toughness of an interface along a thin ductile layer joining elastic solids. *Philos. Mag. A* **70,** 641-656 (1994).
4. Tvergaard, V. & Hutchinson, J. W. Effect of strain-dependent cohesive zone model on predictions of crack growth resistance. *Int. J. Solids Struct.* **33,** 3297-3308 (1996).
5. Muhlstein, C. L., Brown, S. B. & Ritchie, R. O. High-cycle fatigue of single-crystal silicon thin films. *J. Microelectromech. Syst.* **10,** 593-600 (2001).
6. Jung, S. C., Choi, J. W. & Han, Y.-K. Anisotropic volume expansion of crystalline silicon during electrochemical lithium insertion: an atomic level rationale. *Nano Lett.* **12,** 5342-5347 (2012).
7. Li, J. & Dahn, J. R. An in situ x-ray diffraction study of the reaction of Li with crystalline Si. *J. Electrochem. Soc.* **154,** A156-A161 (2007).
